# Reducing Left Ventricular Wall Stress through Aortic Valve Enlargement via Transcatheter Aortic Valve Implantation in Severe Aortic Stenosis

**DOI:** 10.3390/jcm13133777

**Published:** 2024-06-27

**Authors:** Chih-Yao Chiang, Shen-Che Lin, Jung-Cheng Hsu, Jer-Shen Chen, Jih-Hsin Huang, Kuan-Ming Chiu

**Affiliations:** 1 Department of Cardiovascular Surgery, Cardiovascular Center, Far Eastern Memorial Hospital, New Taipei City 220216, Taiwan; femh97737@femh.org.tw (C.-Y.C.); femh76818@femh.org.tw (J.-S.C.); 2 Division of Cardiovascular Surgery, Department of Surgery, School of Medicine, National Defense Medical Center, Taipei 114201, Taiwan; 3 Medical Education Department, Far Eastern Memorial Hospital, New Taipei City 220216, Taiwan; femh97996@femh.org.tw; 4 Department of Cardiology, Cardiovascular Center, Far Eastern Memorial Hospital, New Taipei City 220216, Taiwan; femh85863@femh.org.tw; 5 Department of Applied Cosmetology, LeeMing Institute of Technology, New Taipei City 243083, Taiwan; 6 Department of Electrical Engineering, Yuan Ze University, Taoyuan 320315, Taiwan

**Keywords:** wall stress, ventricular–arterial coupling, low-flow aortic stenosis, transcatheter aortic valve implantation

## Abstract

**Background**: In aortic stenosis, the left ventricle exerts additional force to pump blood through the narrowed aortic valve into the downstream arterial vasculature. Adaptive hypertrophy helps to maintain wall stress homeostasis but at the expense of impaired compliance. Advanced ventricular deformation impacts the extent of functional recovery benefits achieved through transcatheter aortic valve implantation. **Methods and Results**: Subgroups were stratified based on output, with low-flow severe aortic stenosis defined as stroke volume index <35 mL· m^−2^. Before intervention, the low-flow subgroup exhibited worse effective orifice area index and arterial and global impedance, along with thinner wall thickness and larger chamber volume marginally. LV performance, including stroke volume index, ventricular elastance, and ventricular–arterial coupling, were notably inferior, consistent with worse adverse remodeling. Although the effective orifice area index was similarly augmented after TAVI, inferior recovery benefits were noted. Persistently higher wall stress and energy consumption were observed, along with poorer ventricular–arterial coupling. These changes in wall stress showed an inverse relationship with alterations in wall thickness and were proportional to changes in dimension and volume. Additionally, they were proportional to changes in left ventricular end-systolic pressure, pressure–volume area, and ventricular–arterial coupling but inversely related to ventricular end-systolic elastance. **Conclusions**: The study revealed that aortic valve enlargement through transcatheter aortic valve implantation reduces left ventricular wall stress in severe aortic stenosis. The reduced recovery benefits in the low-flow subgroup were evident. Wall stress could serve as a marker of mechanical benefit after the intervention.

## 1. Introduction

Aortic stenosis stands as the most prevalent degenerative valvular disease, often accompanied by comorbidities such as hypertension and atherosclerosis [1]. The left ventricle increases its workload to pump blood through the stenosed aortic valve and into the stiffened arterial vasculature. Prolonged pressure overload leads to distinct structural transformations to preserve adequate output and ventricular–arterial coupling [2]. Adaptive remodeling maintains wall stress homeostasis in response to progressively elevated global afterload, preserving coronary reserve at the cost of increased energy consumption and mechanical efficiency [3]. Concentric hypertrophy results in impaired compliance and diastolic dysfunction, gradually affecting ventricular output [4]. Transcatheter aortic valve implantation (TAVI) enlarges the aortic valve area and reduces afterload, thereby improving left ventricular (LV) performance. This intervention enhances compliance, reduces wall stress and inefficient work, and initiates reverse remodeling and mass regression [4,5]. The extent of pre-existing deformation significantly influences the recovery benefits of hemodynamic and reverse remodeling, thereby determining outcomes [6,7]. This study assessed the immediate effects on hemodynamic recovery by stratifying output flow in severe aortic stenosis. The objectives were twofold: first, to compare differences in geometry and hemodynamics immediately following the intervention, and second, to assess the correlation between hemodynamic variables and the effects of hemodynamic recovery.

## 2. Materials and Methods

### 2.1. Study Population

This study recruited 62 patients who received TAVI for severe AS from January 2016 to December 2023. The high-risk stratification was established according to the calculated Society of Thoracic Surgeons (STS) score >10%. All data were retrieved from the electronic medical records and from clinic visits. The study procedures were performed according to the guidelines stipulated in the Declaration of Helsinki. The study was approved by the Institutional Review Board of Far Eastern Memorial Hospital (approval number 111256-E).

### 2.2. Doppler Echocardiography and TAVI Procedure and Prosthesis 

All patients underwent routine Doppler echocardiography before TAVI, before discharge during the same hospitalization, and during follow-up evaluations at the outpatient department. A single cardiologist conducted all evaluations using an ultrasound system (Philips iE33, Philips Medical Systems, Andover, MA, USA) without inotropic support. All TAVI procedures were performed under general anesthesia, with vascular access achieved either percutaneously or via surgical cutdown through the common femoral artery. Transfemoral placement involved retrograde delivery through arterial access with an introducer sheath, advancing the stented valve crimped onto the delivery balloon under fluoroscopic guidance. The valve was deployed under rapid right ventricular pacing in a sub-coronary location, with assistance from fluoroscopic and transesophageal echocardiography guidance. Acute device success was defined as the adequate technical placement of the valve within the aortic root without fatal complications.

### 2.3. Outcomes of Hospital and Surveillance and Definition of Major Complications 

In-hospital mortality included all-cause deaths occurring during the same hospitalization. Survivors underwent follow-up at the outpatient department and received echocardiographic assessments at our institution. Major adverse cardiovascular events included myocardial ischemia requiring intervention, cerebral thromboembolism, heart failure necessitating hospitalization, and death. The severity of paravalvular leak (PVL) was evaluated using transthoracic echocardiography and classified according to the Valve Academic Research Consortium (VARC)-2 recommendations. Permanent pacemaker implantation was performed in cases of high-degree atrioventricular block or left bundle branch block resulting from direct mechanical compression after prosthesis implantation.

### 2.4. Dimension, Volume, Mass, and Remodel Mode of Left Ventricle

The geometric measurements, including LV end-diastolic diameter (LVEDD), LV end-systolic diameter (LVESD), and LV posterior wall thickness (PWT), were calculated using M-mode echocardiography. LV end-diastolic volume (LVEDV) and end-systolic volume (LVESV) were calculated from the apical two-chamber and four-chamber views using the Teichholz formula or Simpson method. LV mass (LVM) is calculated using the corrected American Society of Echocardiography formula.

$$LV\;mass=0.8\times \left[1.04\times \left({\left(IVST+LVEDD+PWT\right)}^{3}-{LVEDD}^{3}\right)\right]+0.6\;LV\;mass\;index=\frac{LV\;mass}{body\;surface\;area}\;\;\;\;\;\;\;\;Relative\;wall\;thickness=\frac{2\times Posterior\;wall\;thickness}{LV\;end\;diastolic\;dimension}$$

Hypertrophy was defined as a left ventricular (LV) mass index exceeding the cutoff values of ≥95 g∙m^−2^ for females and ≥115 g∙m^−2^ for males. Concentric hypertrophy was characterized by the presence of hypertrophy along with a relative wall thickness (RWT) of ≥0.42, whereas eccentric hypertrophy was identified by hypertrophy accompanied by an RWT <0.42.

### 2.5. Valvular Load, Arterial Load, and Global LV Afterload

LV outflow tract (LVOT) is measured in the parasternal long-axis view on the mid-systole phase. The transaortic flow velocities are measured, and the effective orifice area (EOA) is calculated with a modified Bernoulli equation and continuity equation, then indexed to the body surface area to assess the severity.

$$Effective\;orifice\;area=\frac{{Area}_{LVOT}\times {VTI}_{LVOT}}{{VTI}_{AV}}\;\;\;\;\;Indexed\;effective\;orifice\;area=\frac{effective\;orifice\;area}{body\;surface\;area}$$

Stratification of aortic stenosis severity was defined as severe if EOAI < 0.65 cm^2^/m^2^ and moderate if EOAI ≥ 0.65 cm^2^/m^2^ and ≤0.85 cm^2^/m^2^. The pressure gradient was estimated from the simplified Bernoulli equation. LV stroke work loss was represented by the portion of LV pressure–volume work per stroke lost because of outflow tract obstruction.

$$\Delta Ρ≅4\cdot V_{max}^{2}\;\;\;\;\;\;\;\;\;Stroke\;work\;loss=\frac{mean\;pressure\;gradient}{LV\;end\;systolic\;pressure}\;LV\;end\;systolic\;pressure=\left(0.9\times systolic\;arteria\;pressure\right)+mean\;pressure\;gradient$$

Systemic vascular resistance is the force exerted on circulating blood by the vasculature of the body. Systemic arterial compliance can be estimated using the stroke volume index-to-aortic pulse pressure ratio (SVI/PP) as a surrogate for arterial impedance. The global LV afterload, valvular–arterial impedance (Zva), is the sum of valvular and arterial load divided by SVI. It represents the valvular and arterial factors that oppose ventricular ejection by absorbing the mechanical energy developed by the LV.

$$SVR=\frac{80\times mean\;arterial\;pressure}{cardiac\;output}\;\;\;\;\;\;\;\;\;\;\;SAC=\frac{stroke\;volume\;index}{pulse\;pressure}\;Zva=\frac{\left(systolic\;arterial\;pressure+mean\;transaortic\;pressure\right)}{stroke\;volume\;index}$$

### 2.6. Wall Stress, LV Performance, and Energetics

$$End\;systolic\;meridional\;wall\;stress=\frac{1.33\times (LV\;end\;systolic\;pressure)\times (LV\;end\;systolic\;dimension)}{4h\times (1+\frac{h}{LVESD})}$$

where h is end-systolic LV wall thickness calculated as the mean between LV end-systolic septal thickness and posterior wall thickness [8].

$$Stroke\;volume=\left[\pi \times {\left(\frac{LVOT}{2}\right)}^{2}\right]\times {VTI}_{LVOT}\;\;\;\;\;\;\;\;Stroke\;volume\;index=\frac{stroke\;volume}{body\;surface\;area}$$

The low-flow subgroup was defined as stroke volume index ≤35 mL·m^−2^ [6].

$$Ejection\;fraction=\frac{LV\;end\;diastolic\;volume-LV\;end\;systolic\;volume}{LV\;end\;diastolic\;volume}\;Fraction\;shortening=\frac{LV\;end\;diastolic\;dimension-LV\;end\;systolic\;dimension}{LV\;end\;diastolic\;dimension}$$

Ventricular time-varying elastance: the summation of actin–myosin coupling to counteract afterload [9] (as depicted in central illustration).

Ees: LV end-systolic elastance represents the load-independent index of LV intrinsic contractility and LV end-systolic stiffness [10].

Ea: Arterial elastance represents an integrative index of arterial load.

Ventricular–arterial coupling represents the transfer efficiency from the left ventricle to arterial vasculature.

$$Ea=\frac{LV\;end\;systolic\;pressure}{Stroke\;volume}\;\;\;\;\;\;Ees=\frac{LV\;end\;systolic\;pressure}{end\;systolic\;volume}\;\;\;\;\;\;VA\;coupling=\frac{Ea}{Ees}\;Stroke\;Work\approx Stroke\;volume\times LV\;end\;sytolic\;pressure$$

The approximation of potential energy was represented by a triangle formed by the ESPVR, the end-systolic volume, and the x-axis.

Potential Energy=0.5×LV end systolic volume×LV end systolic pressure

Pressure-volume area = Potential Energy + Stroke Work [11]

Mechanical Efficiency = $\frac{stroke\;work}{PV\;area}$ [12]

Relative change in hemodynamics

$$\mathrm{Change}\;\mathrm{of}\;\mathrm{hemodynamics}=postoperative\;value-preoperative\;value\;Relative\;change\;of\;hemodynamics=\frac{postoperative\;value-preoperative\;value}{preoperative\;value}$$

$$Relative\;change\;of\;hemodynamics=\frac{postoperative\;value-preoperative\;value}{preoperative\;value}$$

### 2.7. Statistical Analysis

Continuous variables are presented as median and 95% confidence interval. The independent samples are tested with the Mann–Whitney test, and the paired samples are tested with the Wilcoxon test. Categorical variables are presented as frequencies and compared with Fisher's exact test. Linear regression analysis identified the independent hemodynamics. All tests are two-tailed, and the level of statistical significance is set at *p* < 0.05. The statistical analyses are performed using MedCalc statistical software V22.021.

## 3. Results

Sixty-two patients underwent transcatheter aortic valve implantation (TAVI) from January 2016 to December 2023. The median surveillance duration was 19 months (95% CI 14 to 21), with males accounting for 47% of the cohort. The transcatheter prosthetic valves used included CoreValve in 8 cases, Evolute-R in 35 cases, Evolute-Pro in 8 cases, and MyVal in 11 cases. CoreValve prostheses were implanted from January 2016 to May 2021, Evolute-R prostheses from April 2017 to August 2022, Evolute-Pro prostheses starting in October 2022, and MyVal prostheses were introduced in January 2023. There were no mortalities during the same hospitalization, and only one patient required reimplantation due to migration. Six patients (10%) required permanent pacemaker implantation due to conduction disturbances, and three patients required ventilator support for more than 48 h. Paravalvular regurgitation was classified as trivial in 22 cases (35.5%) and mild in 11 cases (17.7%). Clinical outcomes revealed 19 cases (30.6%) of major adverse cardiac events (MACEs), with all-cause deaths in 12 cases (19.4%) and cardiac deaths in 8 cases (12.9%). In the low-flow subgroup, out of nine patients, one exhibited left bundle branch block (LBBB), one had more than mild paravalvular leak (PVL), and three experienced acute kidney injury (AKI).

### 3.1. Baseline Characteristics and Operative Results 

Presented in Table 1, the subgroups were stratified based on stroke volume indexed to body surface area, using a cutoff value of 35 mL∙m^−2^. Among comorbidities, higher prevalence rates were observed for hypertension (74%), dyslipidemia (63%), coronary artery disease (58%), and chronic kidney disease (53%). Additionally, atrial fibrillation (23%) and pulmonary hypertension (grade ≥ 2) (32%) were identified. However, no significant differences were found between the subgroups. Concentric hypertrophy was observed in 81% of cases, with no notable discrepancies between subgroups. Systolic dysfunction (LVEF < 55%) was detected in 19% of all participants, 17% in the normal-flow subgroup, and 33% in the low-flow subgroup. Diastolic dysfunction (grade ≥ 2) was present in 50% of all participants, 45% in the normal-flow subgroup, and 78% in the low-flow subgroup.

### 3.2. Comparisons of Geometric and Hemodynamic Parameters following TAVI

After analyzing the entire cohort of 62 patients following TAVI, as outlined in Table 2, several significant findings emerged. Firstly, both wall thickness and relative wall thickness increased. Additionally, there was a reduction in chamber dimensions, including both end-systolic and end-diastolic dimensions. Intracavity volumes showed a decrease in both the end-systolic volume index (ESVI) and end-diastolic volume index (EDVI). Regarding valvular load, an augmented effective orifice area index (EOAI) was achieved, accompanied by a reduction in the transvalvular pressure gradient and stroke work loss. Concerning arterial load, there was a reduction in systemic vascular resistance and arterial elastance, coupled with an increase in systemic arterial compliance. Significant reductions in valvular–arterial impedance, left ventricular end-systolic pressure, and wall stress were observed. In terms of LV performance, improvements were noted in stroke volume index (SVI), fractional shortening, and ejection fraction. However, intrinsic ventricular end-systolic elastance decreased, along with diminished total energy consumption and improved transfer efficiency. As shown in Table 3, the low-flow subgroup exhibited similar alteration trends to the normal-flow subgroup, except for marginal changes in dimension, arterial load, and contractility.

### 3.3. Comparisons of Baseline and Post-TAVI between Subgroups

As depicted in Table 4, the subgroup analysis conducted prior to TAVI revealed several findings. The low-flow subgroup demonstrated marginally thinner wall thickness and increased intracavity volume. It exhibited a smaller effective orifice area index (EOAI) and pronounced stroke work loss. Additionally, heightened arterial impedance and reduced compliance were observed, indicating severe arterial stiffness and elevated global afterload. The LV performance, including stroke volume index (SVI), ventricular end-systolic elastance (Ees), and ventricular–arterial coupling (VAC), was notably worse. These findings indicated a more advanced disease progression in the low-flow subgroup. Similar levels of wall stress and pressure–volume area (PVA) were observed between the subgroups, while lower Ees and marginally reduced LV end-systolic pressure (LVESP) were noted. Following TAVI, the low-flow subgroup still exhibited marginally thinner wall thickness and larger intracavity volume. Although similar effective orifice area index (EOAI), mean pressure gradient (MPG), and stroke work loss, the low-flow subgroup still showed worse arterial impedance and global afterload. In terms of LV performance, this subgroup disclosed a lower stroke volume index, ventricular elastance, and LV end-systolic pressure. Higher levels of wall stress and total energy consumption were observed, along with poorer ventricular–arterial coupling.

### 3.4. Correlation between Relative Changes of Geometry and Hemodynamics

**Figure 1:**
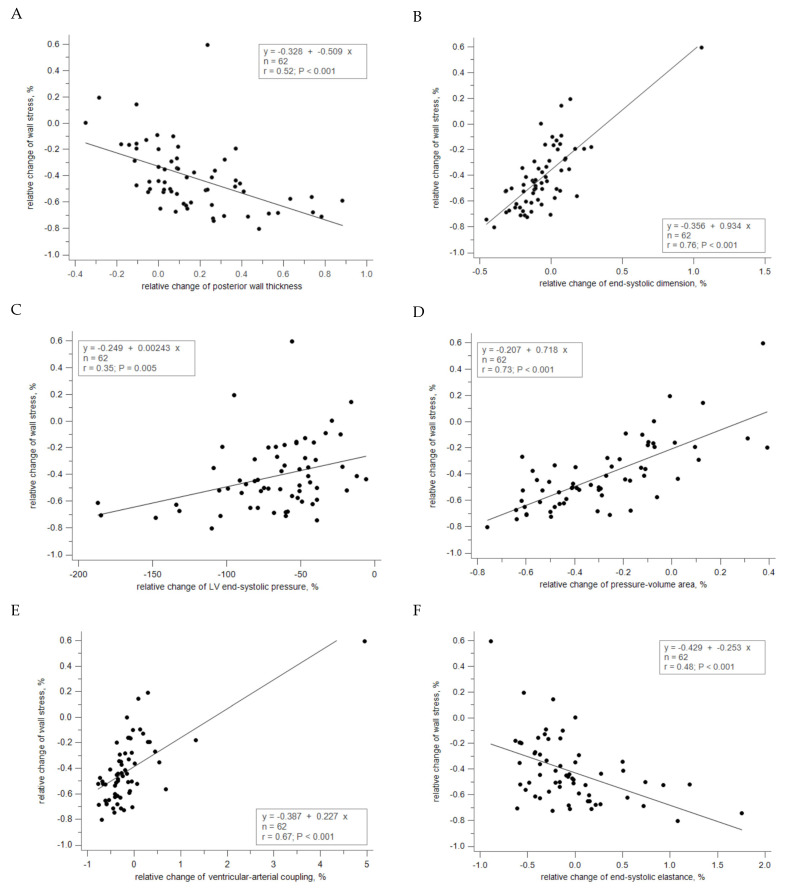
Correlation in alterations of geometry and hemodynamics from baseline to post-TAVI with linear regression. (**A**) The relative change in posterior wall thickness is inverse relation to relative change in wall stress; (**B**) the relative change in end-systolic dimension is proportional to relative change in wall stress; (**C**) the relative change in end-systolic pressure is proportional to relative change in wall stress; (**D**) the relative change in end-systolic elastance is inverse relation to relative change in wall stress; (**E**) the relative change in pressure–volume area is proportional to relative change in wall stress; (**F**) the relative change in pressure–volume area is proportional to relative change in wall stress.

As depicted in Table 5 and Figure 1, the relative changes in EOAI showed an inverse relationship with the relative changes in MPG and SWL in valvular load. Conversely, EOAI displayed a proportional relationship with the relative changes in SAC but an inverse correlation with the relative changes in arterial elastance (Ea) in arterial load. Additionally, EOAI demonstrated an inverse correlation with the relative changes in Zva and LVESP while showing a proportional correlation with SVI. The relative change in systemic arterial compliance showed no significant correlation with geometry but exhibited a proportional relationship with the relative changes in EOAI and SVI. It displayed an inverse correlation with the relative changes in SVR, Ea, Zva, and LVESP. Regarding Zva, its relative changes demonstrated a proportional relationship with the relative changes in MPG, SVR, Ea, LVESP, and Ees. However, it exhibited an inverse correlation with the relative changes in EOAI, SAC, and SVI. On the other hand, the relative changes in left ventricular end-systolic pressure (LVESP) showed no correlation with geometry but displayed an inverse relationship with the relative changes in EOAI and systemic arterial compliance (SAC). Conversely, it exhibited a proportional relationship with the relative changes in mean pressure gradient (MPG) and stroke work loss (SWL) of valvular load, arterial elastance (Ea), and global load. It was also proportional to wall stress, ventricular elastance (Ees), and pressure–volume area (PVA). Additionally, the relative changes in wall stress exhibited an inverse relationship with the relative changes in posterior wall thickness (Figure 1A) and were proportional to changes in end-systolic dimension (Figure 1B) and volume. Moreover, wall stress showed a proportional correlation with the relative changes in LVESP (Figure 1C), PVA (Figure 1D), and ventricular–arterial coupling (Figure 1E), but an inverse relationship with Ees (Figure 1F), fractional shortening, and ejection fraction.

## 4. Discussion

### 4.1. Pathophysiology of Adaptive Hypertrophy to Pressure Overload

**Figure 2:**
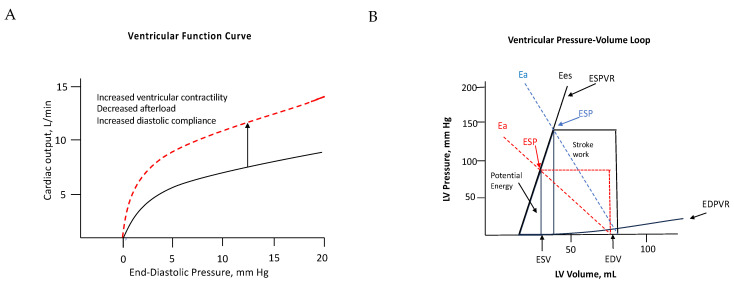
(**A**) The ventricular function curve shifts up and to the left (indicating from black line to red dotted line) with increased ventricular contractility, decreased afterload, and increased compliance. (**B**) TAVI reduces LV end-systolic pressure (LVESP), LV end-systolic volume (LVESV), and LV end-diastolic volume (LVEDV), improving afterload and compliance, ultimately resulting in decreased potential energy and stroke work and increased mechanical efficiency. Ea, arterial elastance; EDPVR, end-diastolic pressure–volume relationship; EDV, LV end-diastolic volume; Ees, LV end-systolic elastance; ESP, LV end-systolic pressure; ESPVR, end-systolic pressure–volume relationship; ESV, end-systolic volume; PE, potential energy; SW, stroke work.

Aortic stenosis is the most prevalent structural valvular disease in the elderly population, often associated with hypertension and arterial sclerosis [1]. The left ventricle pumps blood through a stenosed aortic valve, which leads to an increased workload and higher stroke work loss [13,14,15]. The shear stress caused by turbulent flow triggers an inflammatory reaction and sclerosis in the aortic valve and downstream aorta [16]. The systolic force generated by ventricular contractility propels blood flow downstream, counteracting the dual burdens of valvular and arterial loads [8,17,18,19]. In response to gradually increasing pressure overload, the endomyocardium triggers the NO-cGMP-PKG-Titin signaling pathway and an inflammatory reaction [20]. Adaptive hypertrophy aims to maintain wall stress homeostasis and ensure coronary dilatory reserve, thereby preventing dysfunction in conduction and contractility. Wall stress correlates not only with LV end-systolic pressure but also with dimensions and wall thickness, in accordance with Laplace’s law [8,21]. Decompensated remodeling compromises ventricular output when wall stress progressively elevates during transitional transformation [3,22,23]. According to the Frank–Starling law, LV performance is influenced by afterload, compliance, and contractility (Figure 2) [24]. Maladaptive hypertrophy, accompanied by elevated wall stress, adversely affects contractility due to microvascular compression and limited availability of viable myocardium during the transitional fibrotic process [25,26,27,28]. Structural deformation reflects deterioration characterized by myocyte loss, post-apoptotic replacement fibrosis, and maladaptive hypertrophy [27].

### 4.2. Stratification of Adverse Remodeling by Output Using Stroke Volume Index

The objective of TAVI is to reduce valvular load by enlarging the stenosed aortic valve, thereby improving output. Patients with a stroke volume index below 35 mL∙m^−2^ were identified as the low-flow subgroup [6,26]. It exhibited a higher global load (Zva), primarily due to increased arterial load, combined with a more distended chamber, pronounced hypertrophy, and impaired compliance [17]. Concentric hypertrophy exacerbates microvascular perfusion by increasing intracavity pressure and periarterial interstitial resistance. This compressive force adversely affects subendocardial diastolic perfusion [22]. Ees represents ventricular end-systolic elastance, serving as a surrogate of intrinsic contractility, and it denotes the maximal actin–myosin coupling during ejection. The reduced ventricular elastance suggests a diminished availability of actin–myosin coupling to counteract LV impedance, resulting in lower force to propel blood flow downstream [2,29,30]. Prolonged exposure to pressure overload is associated with post-apoptotic replacement fibrosis, the fibrotic substitution of viable myocardium compromising contractility [31]. Advanced structural deformation due to replacement fibrosis was correlated with geometric modifications and dysfunction [27,28]. 

### 4.3. Diversity in Recovery Benefits by Afterload Reduction through TAVI

The normal-flow subgroup exhibited significant changes in geometric and hemodynamic parameters following the intervention. However, the low-flow subgroup showed only marginal geometric changes and a persistently higher residual volume after ejection. Although the effective orifice area index and stroke work loss were similar among the subgroups, the low-flow subgroup had higher afterload, including Ea and Zva, along with impaired compliance and higher wall stress. Due to previously advanced maladaptation, there were fewer recovery benefits in wall stress and stroke volume in the low-flow subgroup.

### 4.4. Correlation between Hemodynamics and Energetics 

The study examined the relationship between hemodynamic and energetic parameters. Pressure stress activates the endocardium, triggering the NO-cGMP-PKG-Titin signaling pathway and downstream actin–myosin coupling and contractility as an adaptation to progressively increased pressure overload [20]. The cyclic changes in time-varying elastance correlated with the summative forces of actin–myosin coupling to counteract varying afterload [9,29,30]. Ventricular elastance serves as a surrogate for intrinsic contractility, effectively representing the conversion of chemical energy into mechanical work [24]. The LV end-systolic pressure is proportional to ventricular end-systolic elastance (Ees). LV systolic pressure represents the force generated by myocardial contractility to counteract the global load during ejection [17]. Adaptive remodeling manifests as concentric hypertrophy, characterized by the thickening of the ventricular wall and reduced chamber dimensions. This adaptive shape helps to reduce wall stress and counteract the effects of pressure overload. Changes in ventricular elastance were inversely related to variations in wall stress, as improved coronary perfusion enhances contractility [21]. The pressure–volume area, representing the sum of potential energy and stroke work within the pressure–volume relationship, serves as a surrogate of total energy consumption in each contraction cycle. Wall stress is proportional to total energy consumption. The PV loop shift to the left indicates reduced wall stress and improved mechanical efficiency (Central illustration). Ventricular–arterial coupling reflects the effectiveness of pumping blood from the ventricle to the arterial vasculature. Reduced wall stress has been confirmed to be associated with improved ventricular–arterial coupling. TAVI enhances ventricular output by expanding the aortic valve and reducing wall stress, leading to more effective mechanical work with lower energy consumption. This is achieved by reducing end-systolic volume (ESV), end-diastolic volume (EDV), and end-systolic pressure (ESP) and shifting the pressure–volume (PV) loop to the left. It supports increased blood flow to the arterial vasculature by improving ventricular–arterial coupling. Baseline geometric transformation and intrinsic structural changes impact the recovery benefits from the intervention.

### 4.5. Clinical Implication

Adaptive remodeling of the left ventricle during structural evolution aims to maintain wall stress homeostasis, protecting the myocardium from injury due to pressure overload until decompensation occurs. Enlarging the stenosed aortic valve reduces valvular load and improves compliance. Reduction in global afterload and wall stress enhances ventricular–arterial coupling and subsequently improves output. In the low-flow subgroup, functional recovery is less pronounced, likely due to previously advanced maladaptation. Analyzing hemodynamic changes provides insight into the immediate energetic benefits after intervention. 

### 4.6. Study Limitation

This study has several limitations that warrant consideration. Firstly, it is a single-center, non-randomized retrospective cross-sectional study with a limited number of patients, especially in the low-flow subgroup. Secondly, the quality of echocardiographic assessments may have varied due to differences in observer technique and interpretation. Thirdly, the study primarily focused on acute changes in geometry, hemodynamics, and energetics following the intervention. Long-term follow-up with more participants is essential to evaluate the persistence of these results over time.

## 5. Conclusions

Although ventricular performance generally improves following TAVI, the immediate mechanical benefits vary depending on the extent of prior maladaptation. In low-flow severe aortic stenosis, which indicates advanced adverse remodeling, the benefits from recovery of wall stress and ventricular–arterial coupling are reduced. These reduced benefits were verified through TAVI. Ventricular wall stress could serve as an indicator of mechanical benefits.

## Figures and Tables

**Table 1:** Baseline characteristics of the total cohort and subgroups.

Variable	Total (n = 62)	Normal Flow (n = 53)	Low Flow (n = 9)	*p*
Age (y)	81.4 ± 6.5	81.1 ± 6.4	82.7 ± 6.8	0.514
Body surface area	1.56 ± 0.17	1.60 ± 0.17	1.53 ± 0.16	0.465
Sex	29 (47)	26 (49)	3 (33)	0.271
Comorbidity				
Hypertension	46 (74)	40 (75)	6 (67)	0.683
Coronary artery disease	39 (63)	36 (68)	3 (33)	0.066
Dyslipidemia	36 (58)	32 (60)	4 (44)	0.473
Diabetes	24 (39)	22 (42)	2 (22)	0.462
Chronic kidney disease	33 (53)	29 (55)	4 (44)	0.722
Atrial fibrillation	14 (23)	11 (21)	3 (33)	0.409
Cerebrovascular accident	7 (11)	7 (13)	0 (0)	0.580
Pulmonary hypertension, Gr ≥ 2	20 (32)	18 (34)	2 (22)	0.705
Dysfunction				
Systolic, ejection fraction < 55%	12 (19)	9 (17)	3 (33)	0.357
Diastolic, Gr < 2	5 (8)	4 (8)	1 (11)	0.557
Diastolic, Gr ≥ 2	31 (50)	24 (45)	7 (78)	0.147
Remodel mode				
Normal	1 (2)	1 (2)	0 (0)	1.000
Concentric remodel	3 (5)	3 (6)	0 (0)	1.000
Concentric hypertrophy	50 (81)	42 (79)	8 (89)	0.675
Eccentric hypertrophy	8 (13)	7 (13)	1 (11)	1.000

Data are expressed as numbers (percentage, %); mean ± standard deviation.

**Table 2:** Comparisons of acute changes in geometry and hemodynamics between baseline and post-TAVI in total cohort.

	Total (n = 62)				
	Baseline		Post-TAVI		*p*
Geometry					
PW, cm	1.32	1.24, 1.35	1.50	1.40, 1.57	<0.001
RWT, %	0.54	0.52, 0.60	0.66	0.60, 0.72	<0.001
ESD, cm	2.99	2.86, 3.26	2.94	2.67, 3.00	<0.001
EDD, cm	4.75	4.44, 5.04	4.49	4.32, 4.74	0.015
ESVI, mL·m^−2^	22.64	19.56, 26.23	20.35	16.75, 23.12	<0.001
EDVI, mL·m^−2^	65.46	61.85, 74.22	63.12	53.85, 67.13	0.016
Valvular load					
EOAI, cm^2^·m^−2^	0.47	0.43, 0.52	1.12	1.06, 1.25	<0.001
MPG, mmHg	41	36, 46	8	7, 9	<0.001
SWL, %	23.79	21.65, 27.15	6.18	4.87, 6.93	<0.001
Arterial load					
SVR, Kdynes·sec^−1^·cm^−5^	1.36	1.29, 1.50	1.14	1.05, 1.20	<0.001
SAC, mL·mmHg^−1^·m^−2^	0.76	0.67, 0.88	0.84	0.78, 0.93	<0.001
Ea, mmHg·mL^−1^	2.87	2.65, 3.13	1.86	1.70, 2.03	<0.001
Global load					
Zva, mmHg·mL^−1^·m^2^	3.58	3.30, 3.99	2.69	2.46, 2.87	<0.001
ESP, mmHg	208	192, 220	147	138, 151	<0.001
WS, Kdyne·cm^−2^	113.31	98.97, 129.66	63.20	58.69, 68.32	<0.001
LV performance					
SVI, mL·m^−2^	47.11	44.27, 51.90	51.95	48.32, 54.12	<0.001
FS, %	35.77	33.54, 37.61	36.89	34.27, 37.85	0.008
EF, %	65.35	62.32, 67.68	66.79	63.42, 68.39	0.005
Ees, mmHg·mL^−1^	5.64	4.80, 6.52	4.68	4.01, 5.07	0.002
Energetic					
PV area, cJ	152.99	145.28, 188.16	106.90	93.93, 119.40	<0.001
VA coupling, ratio	0.50	0.43, 0.68	0.40	0.32, 0.47	<0.001

Data are expressed as median, 95% confidence interval. Abbreviation: PW, posterior wall thickness; RWT, relative wall thickness; ESD, end-systolic dimension; EDD, end-diastolic dimension; ESVI, end-systolic volume index; EDVI, end-diastolic volume index; EOAI, effective orifice area index; MPG, mean transaortic pressure gradient; SWL, stroke work loss; SVR, systemic vascular resistance; SAC, systemic arterial compliance; Ea, arterial elastance; Zva, valvular–arterial impedance; ESP, end-systolic pressure; WS, wall stress; SVI, stroke volume index; FS, fractional shortening; EF, left ventricular ejection fraction; Ees, left ventricular end-systolic elastance; PV area, pressure–volume area; VA coupling, ventricular–arterial coupling; cJ, centi Joule.

**Table 3:** Comparisons of acute changes in geometry and hemodynamics between baseline and post-TAVI in subgroups.

	Normal Flow (n = 53)					Low Flow (n = 9)				
	Baseline		Post-TAVI		*p*	Baseline		Post-TAVI		*p*
Geometry										
PW, cm	1.32	1.25, 1.39	1.52	1.42, 1.57	<0.001	1.22	1.05, 1.45	1.40	1.12, 1.56	0.176
RWT, %	0.54	0.52, 0.62	0.68	0.60, 0.73	<0.001	0.52	0.42, 0.61	0.61	0.49, 0.70	0.164
ESD, cm	2.96	2.77, 3.24	2.95	2.61, 3.00	0.002	3.25	2.96, 3.87	2.92	2.83, 3.42	0.192
EDD, cm	4.73	4.39, 5.17	4.43	4.30, 4.74	0.038	4.80	4.38, 5.27	4.58	4.21, 5.02	0.164
ESVI, mL·m^−2^	22.02	19.03, 25.95	19.93	15.55, 23.10	0.001	27.04	22.46, 40.89	21.28	19.59, 30.86	0.301
EDVI, mL·m^−2^	63.96	57.21, 78.93	59.64	51.72, 67.89	0.033	70.65	65.07, 81.26	63.59	54.58, 76.05	0.250
Valvular load										
EOAI, cm^2^·m^−2^	0.49	0.46, 0.53	1.12	1.06, 1.28	<0.001	0.30	0.26, 0.37	1.07	0.88, 1.38	0.004
MPG, mmHg	41	36, 46	8	7, 9	<0.001	44	30, 59	7	4, 15	0.008
Stroke work loss, %	23.08	21.05, 25.64	6.11	4.88, 7.00	<0.001	29.73	20.69, 33.26	6.25	3.31, 12.42	0.004
Arterial load										
SVR, Kdynes·sec^−1^·cm^−5^	1.33	1.17, 1.41	1.08	1.03, 1.18	0.002	1.75	1.63, 2.04	1.32	1.20, 1.80	0.060
SAC, mL·mmHg^−1^·m^−2^	0.78	0.70, 0.91	0.86	0.79, 0.96	<0.001	0.60	0.45, 0.97	0.78	0.61, 1.12	0.098
Ea, mmHg·mL^−1^	2.76	2.51, 2.94	1.75	1.65, 1.90	<0.001	4.10	3.60, 5.22	2.27	2.23, 2.58	0.004
Global load										
Zva, mmHg·mL^−1^·m^2^	3.50	3.26, 3.73	2.52	2.38, 2.76	<0.001	5.41	4.45, 7.00	3.22	3.01, 3.93	0.004
ESP, mmHg	210	202, 220	149	138, 153	<0.001	185	168, 231	129	118, 168	0.004
Wall stress, Kdyne·cm^−2^	115.00	91.12, 129.23	62.18	58.39, 68.01	<0.001	105.17	90.70, 181.89	70.37	50.28, 100.05	0.004
LV performance										
SVI, mL·m^−2^	50.89	46.36, 54.42	53.14	50.77, 57.30	0.010	28.42	24.46, 32.22	35.45	32.93, 49.24	0.004
FS, %	35.92	34.08, 37.65	37.24	35.29, 38.50	0.017	31.58	18.32, 41.47	32.35	29.87, 37.26	0.359
EF, %	65.52	63.25, 67.78	67.67	64.35, 68.73	0.010	59.47	37.80, 72.07	60.87	57.14, 67.27	0.301
Ees, mmHg·mL^−1^	6.16	4.89, 6.94	4.84	4.13, 5.57	0.002	4.65	2.99, 7.29	3.94	3.47, 4.76	0.426
Energetic										
PV area, cJ	155.27	145.78, 189.34	105.21	94.07, 116.07	<0.001	145.88	108.44, 202.47	118.76	77.95, 141.94	0.027
VA coupling, ratio	0.46	0.42, 0.51	0.36	0.31, 0.42	<0.001	0.89	0.70, 1.68	0.61	0.55, 0.69	0.004

Data are expressed as median, 95% confidence interval. Abbreviations are illustrated in Table 2.

**Table 4:** Comparisons of geometry and hemodynamics between subgroups in baseline and post-TAVI.

	Baseline					Post-TAVI				
	Normal Flow (n = 53)		Low Flow (n = 9)		*p*	Normal Flow (n = 53)		Low Flow (n = 9)		*p*
Geometry										
PW, cm	1.32	1.25, 1.39	1.22	1.05, 1.45	0.379	1.52	1.42, 1.57	1.40	1.12, 1.56	0.093
RWT, %	0.54	0.52, 0.62	0.52	0.42, 0.61	0.418	0.68	0.60, 0.73	0.61	0.49, 0.70	0.153
ESD, cm	2.96	2.77, 3.24	3.25	2.96, 3.87	0.184	2.95	2.61, 3.00	2.92	2.83, 3.42	0.348
EDD, cm	4.73	4.39, 5.17	4.80	4.38, 5.27	0.742	4.43	4.30, 4.74	4.58	4.21, 5.02	0.803
ESVI, mL·m^−2^	22.03	19.03, 25.95	27.04	22.46, 40.89	0.212	19.93	15.55, 23.10	21.28	19.59, 30.86	0.171
EDVI, mL·m^−2^	63.96	57.21, 78.93	70.65	65.07, 81.26	0.466	59.64	51.74, 67.89	63.59	54.58, 76.05	0.682
Valvular load										
EOAI, cm^2^·m^−2^	0.49	0.46, 0.53	0.30	0.26, 0.37	<0.001	1.12	1.06, 1.28	1.07	0.88, 1.38	0.267
MPG, mmHg	41	36, 46	44	30, 59	0.749	8	7, 9	7	4, 15	0.779
Stroke work loss, %	23.08	21.05, 25.64	29.73	20.69, 33.26	0.034	6.11	4.88, 7.00	6.25	3.31, 12.42	0.960
Arterial load										
SVR, Kdynes·sec^−1^·cm^−5^	1.33	1.17, 1.41	1.75	1.63, 2.04	<0.001	1.08	1.03, 1.18	1.32	1.20, 1.80	0.004
SAC, mL·mmHg^−1^·m^−2^	0.78	0.70, 0.91	0.60	0.45, 0.97	0.039	0.86	0.79, 0.96	0.78	0.61, 1.12	0.037
Ea, mmHg·mL^−1^	2.76	2.51, 2.94	4.10	3.60, 5.22	<0.001	1.75	1.65, 1.90	2.27	2.23, 2.58	<0.001
Global load										
Zva, mmHg·mL^−1^·m^2^	3.50	3.26, 3.73	5.41	4.45, 7.00	<0.001	2.52	2.38, 2.76	3.22	3.01, 3.93	0.001
LVESP, mmHg	210	202, 220	185	168, 231	0.131	149	138, 153	129	118, 168	0.522
WS, Kdyne·cm^−2^	115.00	91.12, 129.23	115.17	90.70, 181.89	0.803	62.18	58.39, 68.01	70.37	50.28, 100.05	0.034
LV performance										
SVI, mL·m^−2^	50.89	46.36, 54.42	28.42	24.46, 32.22	<0.001	53.14	50.77, 57.30	35.45	32.93, 49.24	<0.001
FS, %	35.92	34.08, 37.65	31.58	18.32, 41.47	0.259	37.24	35.29, 38.50	32.35	29.87, 37.26	0.099
EF, %	65.52	63.25, 67.78	59.47	37.80, 72.07	0.259	67.67	64.35, 68.73	60.87	57.14, 67.27	0.103
Ees, mmHg·mL^−1^	6.16	4.89, 6.94	4.65	2.99, 7.29	0.029	4.84	4.13, 5.57	3.94	3.47, 4.76	0.031
Energetic										
PV area, cJ	155.27	145.78, 189.34	155.88	108.44, 202.47	0.668	105.2	94.07, 116.07	118.76	77.95, 141.94	0.032
VA coupling, ratio	0.46	0.42, 0.51	0.89	0.70, 1.68	0.001	0.36	0.31, 0.42	0.61	0.55, 0.69	0.002

Data are expressed as median, 95% confidence interval. Abbreviations are illustrated in Table 2.

**Table 5:** Correlation between relative change in hemodynamics, load, wall stress, and energetics.

	EOAI, %			SAC, %			Zva, %			LVESP, %			WS, %		
	β	γ	*p*	β	γ	*p*	β	γ	*p*	β	γ	*p*	β	γ	*p*
Geometry															
PWT, %	0.158	0.04	0.770	−0.277	0.15	0.220	0.120	0.15	0.254	0.045	0.10	0.455	−0.509	0.52	<0.001
RWT, %	−0.059	0.02	0.873	−0.165	0.14	0.283	0.115	0.21	0.105	0.027	0.09	0.505	−0.461	0.69	<0.001
ESD, %	0.079	0.02	0.906	−0.058	0.03	0.837	−0.054	0.05	0.680	−0.020	0.03	0.788	0.934	0.76	<0.001
EDD, %	0.329	0.04	0.744	−0.088	0.03	0.836	−0.157	0.10	0.424	−0.012	0.01	0.913	1.307	0.71	<0.001
ESVI, %	0.013	0.01	0.944	−0.005	0.01	0.942	−0.006	0.02	0.870	−0.003	0.02	0.894	0.238	0.70	<0.001
EDVI, %	0.127	0.04	0.763	−0.003	0.00	0.988	−0.062	0.10	0.447	−0.001	0.00	0.991	0.545	0.71	<0.001
Valvular load															
EOAI, %	−	−	−	0.156	0.37	0.003	−0.090	0.46	<0.001	−0.039	0.35	0.005	−0.033	0.14	0.283
MPG, %	−2.360	0.37	0.003	−0.125	0.05	0.719	0.351	0.28	0.027	0.377	0.53	<0.001	0.276	0.18	0.155
SWL, %	−2.207	0.37	0.003	0.149	0.06	0.647	0.243	0.21	0.103	0.279	0.42	<0.001	0.193	0.14	0.290
Arterial load															
SVR, %	−0.796	0.23	0.072	−0.887	0.61	<0.001	0.384	0.57	<0.001	0.040	0.10	0.421	−0.013	0.02	0.903
SAC, %	0.881	0.37	0.003	−	−	−	−0.366	0.79	<0.001	−0.090	0.34	0.007	−0.050	0.09	0.491
Ea, %	−2.742	0.52	<0.001	−1.651	0.74	<0.001	1.016	0.98	<0.001	0.332	0.56	<0.001	0.145	0.11	0.374
Global load															
Zva, %	−2.358	0.46	<0.001	−1.697	0.79	<0.001	−	−	−	0.270	0.47	<0.001	0.091	0.07	0.563
ESP, %	−3.155	0.35	0.005	−0.128	0.34	0.007	0.833	0.47	<0.001	−	−	−	0.706	0.33	0.009
WS, %	−0.584	0.14	0.283	−0.158	0.09	0.491	0.062	0.07	0.563	0.155	0.33	0.009	−	−	−
Systolic															
SVI, %	1.548	0.46	<0.001	1.035	0.73	<0.001	−0.478	0.72	<0.001	0.006	0.02	0.900	0.011	0.01	0.917
FS, %	0.037	0.01	0.938	0.012	0.01	0.953	0.002	0.00	0.980	0.038	0.09	0.463	−0.301	0.35	0.005
EF, %	−0.150	0.03	0.790	−0.075	0.04	0.750	0.115	0.14	0.295	0.055	0.11	0.374	−0.400	0.39	0.002
Ees, %	−0.431	0.19	0.129	−0.166	0.18	0.166	0.151	0.35	0.006	0.093	0.38	0.002	−0.253	0.48	<0.001
Energetic															
PV area, %	−0.219	0.05	0.681	−0.143	0.08	0.523	0.048	0.06	0.644	0.155	0.33	0.009	0.718	0.73	<0.001
VA coupling, %	−0.098	0.07	0.599	−0.106	0.18	0.172	0.043	0.15	0.236	−0.004	0.02	0.858	0.227	0.67	<0.001

Abbreviations are illustrated in Table 2.

## Data Availability

The data presented in this study are available on request from the corresponding author.

## Abbreviations

AS	Aortic Valve Stenosis
EOAI	Effective Orifice Area Index
Ea	Arterial Elastance
Ees	End-Systolic Ventricular Elastance
LVESP	Left Ventricular End-Systolic Pressure
PVA	Pressure–Volume Area
RWT	Relative Wall Thickness
SAC	Systemic Arterial Compliance
SWL	Stroke Work Loss
SVI	Stroke Volume Index
SVR	Systemic Vascular Resistance
TAVI	Transcatheter Aortic Valve Implantation
VAC	Ventricular–Arterial Coupling
Zva	Valvular–Arterial Impedance
